# Barriers to health care among rural adults by disability status

**DOI:** 10.1111/jrh.70086

**Published:** 2025-10-20

**Authors:** Alexis Swendener, Mariana Tuttle, Ingrid Jacobson, Lisa I. Iezzoni, Robert Barclay, Carrie Henning‐Smith

**Affiliations:** ^1^ Division of Health Policy and Management University of Minnesota School of Public Health Minneapolis Minnesota USA; ^2^ Health Policy Research Center Mongan Institute Massachusetts General Hospital Department of Medicine Harvard Medical School Boston Massachusetts USA

**Keywords:** access to health care, delaying health care, people with disabilities

## Abstract

**Purpose:**

Access to health care supports both individual and population health. Ample research demonstrates access barriers faced by rural residents and people with disabilities; however, less research has examined access barriers for rural residents by disability status or explored differences across multiple types of access barriers. This brief report addresses this gap by examining 11 financial and nonfinancial barriers to accessing health care among rural adults by disability status.

**Methods:**

Using nationally representative data from the 2022 National Health Interview Survey and focusing on rural adults (n = 4,703), we conducted bivariate and multivariate logistic regression analyses comparing 11 separate access barriers by disability status and generated adjusted predicted probabilities of experiencing these barriers, controlling for sociodemographic characteristics.

**Findings:**

Overall, compared to those without disabilities, rural people with disabilities had significantly higher adjusted predicted probabilities of 8 of the 11 access barriers. These include delaying multiple types of care due to cost, not being able to afford prescriptions, and delaying care due to facility hours, insurance acceptance, transportation, and travel time. Rural people with disabilities were, however, more likely than their nondisabled counterparts to report having a usual place for care.

**Conclusions:**

Rural individuals with disabilities face more barriers to care than their peers without disabilities, including delaying care, which can potentially worsen health outcomes. Our findings provide important information for policymakers to improve access to care at the intersection of rurality and disability.

## INTRODUCTION

Access to health care supports both individual and population health. Multiple factors affect access to various health care services, including financial and nonfinancial factors. Conceptualized as the 5 A's of access,^1^, ^2^ these factors include: affordability, or direct costs of care; availability of services provided; accommodation of needs of patients; acceptability of the service; and accessibility in service location, distance/travel time, and transportation.^1^, ^2^ Disparities in access to care are important to understand as a component of addressing population health. Barriers to accessing care are not equally distributed across population groups, and are impacted by social drivers of health,^3^, ^4^ including geographic location and disability status.

Rural residents experience poorer health outcomes, including higher rates of underlying health problems and chronic conditions, and a higher risk of the leading causes of death.^5^, ^6^, ^7^ Rural residents also face unique health care access barriers. For instance, previous research has identified rural‐specific transportation‐related barriers, including limited public transportation, reliance on private vehicles with associated maintenance and fuel costs, and longer distances and travel time to medical facilities.^8^, ^9^ Rural areas also face shortages of medical providers and increases in facility closures, further limiting access to care.^10^, ^11^

For people with disabilities, health disparities include higher mortality rates and poorer health outcomes compared to people without disabilities, attributable not just to underlying conditions or impairments but structural barriers to health and health care access.^12^, ^13^ With diversity across severity and type of disability requiring differing and sometimes greater needs for care, challenges accessing care are especially problematic for people with disabilities. Previous research finds that people with disabilities face numerous barriers to accessing health care.^12^, ^14^ Barriers include a lack of accessible transportation, a lack of accessible facilities, lower satisfaction with care, as well as increased financial burdens among people with disabilities.^15^, ^16^, ^17^ Access to care is especially important to examine at the intersections of these groups, as overall rates of disability are higher in rural areas,^18^, ^19^ making better access to health care for people with disabilities one way to improve population health in rural areas.

Although research has examined geographic differences in health care access barriers,^3^ less research has examined these differences across multiple types of access barriers or among rural residents by disability status.^20^, ^21^ This brief report addresses this gap by examining 11 separate financial and nonfinancial access to care barriers among a nationally representative sample of rural adults and focusing on differences by disability status.

## METHODS

### Data and sample

This study uses data from the 2022 National Health Interview Survey (NHIS), an annual nationally representative survey of the civilian, noninstitutionalized US population, accessed through IPUMS Health Surveys.^22^ The 2022 data was selected because it included additional questions on access to care. Analysis was limited to rural adults (18+) with complete information on sample sociodemographics (n = 4,703). To identify rural residents, we used the 2013 NCHS Urban‐Rural Classification Scheme, which defines all nonmetropolitan counties as rural.^23^

### Measures

The 2022 NHIS asked respondents about 11 barriers to accessing care, 4 financial and 7 nonfinancial barriers. Three questions asked whether costs had delayed care in the past year, with separate questions for medical care, mental health care, and dental care. Another question asked about not getting needed prescription medications because of costs. The 7 nonfinancial barrier questions asked respondents if they had delayed or not received care due to: being unable to get to a facility when it was open; being unable to find a doctor that accepts respondent's insurance; lack of appointment availability; too lengthy of travel time to a facility; respondent's busy schedule; lack of transportation; and not having a usual place for medical care.

Disability status was measured in the NHIS using the Washington Group Short Set (WG‐SS) Composite Disability Indicator,^24^ where respondents were defined as having a disability if they report having “a lot of difficulty” or “cannot do at all” any of the following 6 functions: remembering or concentrating, washing or dressing, communicating in usual language, walking or climbing steps, hearing, or vision.^25^

As neither rural nor disability are monolithic categories and access to care is also impacted by structural factors, other sociodemographic variables associated with access were included in adjusted analyses, including gender (male or female), age, race and ethnicity (non‐Hispanic Black, non‐Hispanic White, Hispanic, American Indian/Alaska Native, and Other), household income (as a ratio to the federal poverty threshold ranging from below 100% to 500+% above), US Census region (Northeast, Midwest, South, and West), educational attainment (less than a high school [HS] diploma, HS diploma/equivalent, some college/2‐year degree, and bachelor's degree or more), marital status (married or unmarried), and employment status (employed during the last week or not). We categorized health insurance status as private, dual‐eligible (both Medicare and Medicaid), Medicare Advantage, traditional Medicare (no Advantage), Medicaid only, other government insurance including Veterans Affairs coverage, uninsured, and unknown.^19^

### Analysis

We conducted bivariate chi‐square analyses comparing rural adults with and without disabilities by sociodemographic characteristics. We then built 11 multivariate logistic regression models to examine each access barrier, adjusting for sociodemographic characteristics, and obtained adjusted predicted probabilities of having each access barrier by disability status. We used Stata 18 and employed NHIS survey weights to account for the complex sample design and to generate nationally representative estimates.

## RESULTS

Table 1 reports the overall rural sample sociodemographics and bivariate comparisons of sociodemographic characteristics between rural respondents with and without disabilities. Overall, 13% of rural residents reported disabilities. Compared to rural people without disabilities, rural people with disabilities were more likely to report having 1 or more access barriers (51.9% vs 38.6%, *P*<.001) and reported a higher average number of access barriers (1.2 vs 0.7, *P*<.001). Examining sociodemographics, compared to people without disabilities, people with disabilities were more likely to be older, have lower incomes, be enrolled in public insurance programs, and less likely to be non‐Hispanic White, have a bachelor's degree, be employed, be married, and be uninsured or have private insurance. More than twice the proportion of rural people with disabilities reported income below the poverty level (25.1% vs 11.6%, *P*<.001) compared to their counterparts without disabilities. Higher proportions of people with disabilities had less than a high school diploma (22.6% vs 12.1%, *P*<.001), and lower proportions had a bachelor's degree (9.1% vs 18.1%, *P*<.001) compared to people without disabilities. Nearly half of rural people with disabilities live in the South, and almost one‐quarter reside in the Midwest.

**TABLE 1:** Rural sample sociodemographic characteristics by disability status.

	All	People with disabilities	People without disabilities	
	Mean/weighted %	Mean/weighted %	Mean/weighted %	*P*‐value
People with disabilities	13.0%			
Avg # of access to care barriers (0‐11)	0.8	1.2	0.7	*P*<.001
Had 1 or more access barrier	40.4%	51.9%	38.6%	*P*<.001
Female	50.2%	52.6%	49.8%	*P* = .29
Age				*P*<.001
18‐24	9.9%	3.2%	10.9%	
25‐34	14.2%	4.8%	15.6%	
35‐44	13.9%	9.9%	14.5%	
45‐54	16.7%	15.3%	17.0%	
55‐64	19.4%	26.5%	18.3%	
65+	25.9%	40.4%	23.7%	
Race and ethnicity				*P* = .04
Non‐Hispanic White	80.9%	78.2%	81.3%	
Hispanic	6.0%	5.0%	6.1%	
Non‐Hispanic Black	7.1%	9.4%	6.8%	
American Indian/Alaska Native	4.4%	6.6%	4.1%	
Other	1.7%	0.9%	1.8%	
Poverty status				*P*<.001
<100% federal poverty level	13.3%	25.1%	11.6%	
100%‐199% federal poverty level	23.5%	31.0%	22.4%	
200%‐299% federal poverty level	19.8%	18.1%	20.0%	
300%‐399% federal poverty level	14.7%	11.0%	15.3%	
400%‐499% federal poverty level	11.6%	7.0%	12.3%	
500+% federal poverty level	17.1%	7.8%	18.5%	
Region				*P* = .02
Northeast	11.0%	11.1%	10.9%	
Midwest	32.9%	23.5%	34.3%	
South	41.1%	48.1%	40.1%	
West	15.0%	17.2%	14.7%	
Education				*P*<.001
Less than HS	13.5%	22.6%	12.1%	
High school/GED	36.7%	37.6%	36.6%	
Some college	32.9%	30.7%	33.2%	
Bachelor's or more	17.0%	9.1%	18.1%	
Married	63.2%	53.4%	64.7%	*P*<.001
Employed	55.5%	20.0%	60.9%	*P*<.001
Health insurance				*P*<.001
Private	54.4%	32.0%	57.8%	
Dual eligible	1.8%	6.4%	1.2%	
Medicare advantage	7.4%	12.1%	6.7%	
Medicare only	3.7%	4.8%	3.5%	
Medicaid only	15.1%	26.4%	13.4%	
Other govt insurer	7.0%	13.8%	6.0%	
Uninsured	10.2%	3.8%	11.2%	
Unknown	0.4%	0.6%	0.4%	
N (weighted %)	4,703 (100.0%)	588 (13.0%)	3,485 (87.0%)	

Table 2 shows the predicted probabilities of having access barriers among rural residents by disability status, adjusting for covariates. Overall, rural people with disabilities had significantly higher adjusted predicted probabilities of 8 of the 11 individual access barriers compared to their counterparts without disabilities, including all 4 of the financial barriers. Among the largest probabilities overall and largest differences by disability status, rural people with disabilities were almost 8% more likely to report delaying medical care due to cost (12.3% vs 4.7%, *P* =  .001) and delaying dental care due to cost (22.6% vs 14.7%, *P*<.001) compared to those without disabilities. Rural people with disabilities were more likely to report not being able to afford prescription medication (8.9% vs 3.5%, *P* =  .002) compared to those without disabilities.

**TABLE 2:** Adjusted predicted probability of having access to care barriers among rural residents by disability status.

	Rural people with disabilities	Rural people without disabilities	*P*‐value
**Financial barriers**			
Delayed medical care due to cost	12.3%	4.7%	*P* = .001
Delayed mental health care due to cost	4.5%	1.5%	*P* = .01
Delayed dental care due to cost	22.6%	14.7%	*P*<.001
Could not afford prescription medication	8.9%	3.5%	*P* = .002
**Nonfinancial barriers**			
Delayed medical care due to hours open	5.4%	2.8%	*P* = .04
Delayed medical care due to insurance acceptance	6.3%	2.0%	*P* = .01
Delayed medical care due to appointment unavailability	10.3%	7.1%	*P* = .07
Delayed medical care due to length of travel time to facility	3.7%	1.3%	*P* = .01
Delayed medical care due to busy schedule	7.9%	6.9%	*P* = .59
Delayed medical care due to lack of transportation	8.7%	3.2%	*P*<.001
Does not have usual place for medical care	2.4%	4.9%	*P* = .003

*Note*: Controlling for: sex, age, race and ethnicity, poverty ratio, Census region, education, marital status, employment status, and health insurance status.

Among the 7 nonfinancial barriers, rural people with disabilities were more likely to report 4 of these compared to their nondisabled counterparts. Rural people with disabilities were more likely to delay care due to lack of transportation (8.7% vs 3.2%, *P*<.001); due to facility hours (5.4% vs 2.8%, *P* =  .04); insurance acceptance (6.3% vs 2.0%, *P* =  .01); and length of travel time to the facility (3.7% vs 1.3%, *P* =  .01), adjusting for covariates. However, we found no significant difference in delaying care due to a busy schedule, with just under 7%‐8% of rural people with and without disabilities having reported this barrier. We also found no significant difference in adjusted probability of delaying care due to appointment unavailability, with over 10% and 7% of rural people with and without disabilities reporting this barrier (*P* =  .07). Notably, rural people without disabilities were more likely not to have a usual place for care compared to their counterparts with disabilities (4.9% vs 2.4%, *P* =  .003).

## DISCUSSION

Overall, we found that rural people with disabilities were more likely than their nondisabled counterparts to experience barriers to accessing care across 8 of 11 barriers considered. These include delaying multiple types of care due to cost, not being able to afford prescriptions, as well as delaying care due to issues with facility hours, insurance acceptance, transportation, and travel time. As delaying care can worsen health outcomes, these findings have negative implications for rural population health generally, which are exacerbated for rural people with disabilities.

Despite facing greater barriers to accessing health care overall, we found that rural people with disabilities were more likely to report having a usual source of care than their rural counterparts without disabilities. This finding may reflect a greater need for ongoing medical management among people with disabilities, and they may be more likely to establish continuous care relationships. In turn, having an ongoing relationship with a provider may help that provider to tailor treatment recommendations to best meet individuals’ needs and personal circumstances to reduce barriers to care.

These results have implications for public policy. We find that rural people with disabilities were more likely to report all 4 financial cost‐related barriers, and this suggests that they may lack adequate insurance coverage, or face higher out‐of‐pocket costs due to more complex health conditions than the general rural population.^26^ Many individuals with disabilities have limited incomes, which likely impacts each of these access barriers and which may require more generous employment benefits and public insurance policies to ensure that they are adequate to meet individuals’ basic needs.

Our results also show that barriers related to transportation and travel time are more common among those with disabilities, highlighting a need for accessible clinics and medical facilities close to where people with disabilities live and with expanded Americans with Disabilities Act‐accessible public or medical transportation options available to those in rural areas.^27^ As rural areas already face shortages in medical providers and specialty care, these nonfinancial barrier disparities highlight how policies to improve access to care may need to account for rural‐specific indirect care costs that people with disabilities experience.^11^ A wide variety of policies already address access to care for rural residents and/or for people with disabilities, all of which could be expanded to address the barriers we examined. These include bolstering public insurance programs and their related benefits (eg, transportation); expanding nonemergency medical transportation, mobile clinics, and telehealth infrastructure; and subsidies to address cost‐sharing and medication costs for rural residents with disabilities.^28^, ^29^, ^30^, ^31^

### Limitations

Findings from this study should be understood in light of their limitations. In particular, we rely on self‐reports of barriers to care, which may suffer from recall bias. We acknowledge the limitations of the WG‐SS disability measure used in the NHIS likely underestimates the size of the population with disabilities compared to measures in other national surveys (such as the American Community Survey), in addition to other types of disabilities that may not be captured by this measure.^25^ As rural populations have higher rates of disability compared to urban populations, it is increasingly important to have accurate measures of the multifaceted nature of disability in order to accurately allocate disability services^32^ to promote rural health equity. Finally, while we are able to identify differences in barriers to care by disability status, we cannot examine all the reasons that people experience those barriers. This should serve as the starting point for future research in this area, including research that further explores differences by age (eg, 65+ and under 65) and sociodemographic characteristics.

## CONCLUSIONS

There is ample research demonstrating barriers to care faced by rural residents and people with disabilities. This study builds on both of those bodies of literature by illuminating barriers faced by rural residents with disabilities. We find that, for 8 of 11 measures, living with a disability is associated with higher rates of barriers to care. These findings provide important information for how to improve access to care at the intersection of rurality and disability.

## CONFLICT OF INTEREST STATEMENT

The authors report there are no competing interests to declare.
